# Female sex reduces the risk of hospital-associated acute kidney injury: a meta-analysis

**DOI:** 10.1186/s12882-018-1122-z

**Published:** 2018-11-08

**Authors:** Joel Neugarten, Ladan Golestaneh

**Affiliations:** Department of Medicine, Nephrology Division, Montefiore Medical Center, Albert Einstein College of Medicine, 111 E. 210 St, Bronx, NY 10467 USA

**Keywords:** Acute kidney injury, Gender, Meta-analysis, Systematic review, Acute renal failure

## Abstract

**Background:**

Female sex has been included as a risk factor in models developed to predict the development of AKI. In addition, the commentary to the Kidney Disease Improving Global Outcomes Clinical Practice Guideline for AKI concludes that female sex is a risk factor for hospital-acquired AKI. In contrast, a protective effect of female sex has been demonstrated in animal models of ischemic AKI.

**Methods:**

To further explore this issue, we performed a meta-analysis of AKI studies published between January, 1978 and April, 2018 and identified 83 studies reporting sex-stratified data on the incidence of hospital-associated AKI among nearly 240,000,000 patients.

**Results:**

Twenty-eight studies (6,758,124 patients) utilized multivariate analysis to assess risk factors for hospital-associated AKI and provided sex-stratified ORs. Meta-analysis of this cohort showed that the risk of developing hospital-associated AKI was significantly greater in men than in women (OR 1.23 (1.11,1.36). Since AKI is not a single disease but instead represents a heterogeneous group of disorders characterized by an acute reduction in renal function, we performed subgroup meta-analyses. The association of male sex with AKI was strongest among studies of patients who underwent non-cardiac surgery. Male sex was also associated with AKI in studies which included unselected hospitalized patients and in studies of critically ill patients who received care in an intensive care unit. In contrast, cardiac surgery-associated AKI and radiocontrast-induced AKI showed no sexual dimorphism.

**Conclusions:**

Our meta-analysis contradicts the established belief that female sex confers a greater risk of AKI and instead suggests a protective role.

## Background

Sexual dimorphism is a well-established feature of chronic progressive kidney disease [1]. Although less well recognized, sexual dimorphism has also been established in the development of ischemic acute kidney injury (AKI) [2]. Animal models have consistently demonstrated that female sex is protective in the development of AKI after ischemia-reperfusion injury [2–14]. Despite these experimental observations, it has been suggested that the direction of sexual dimorphism is reversed in humans with AKI. Female sex has been included as a risk factor in models developed to predict the risk of AKI associated with cardiac surgery, aminoglycoside nephrotoxicity, rhabdomyolysis and radio-contrast administration [15–18]}. The commentary to the Kidney Disease Improving Global Outcomes (KDIGO) Clinical Practice Guideline for Acute Kidney Injury (arguably the most authoritative commentary in the field) states that female sex is among the “shared susceptibility factors” that confer a higher risk of AKI [19]. This conclusion is based on observations that female sex is associated with a higher risk for AKI after cardiac surgery and after the administration of radio-contrast or aminoglycosides. On this basis, the commentary concludes that, “contrary to most chronic kidney disease disorders, it is the female gender that carries a higher risk for AKI.” This conclusion, however, is qualified by the observation that males predominate in reports of AKI complicating infections with HIV, malaria, leptospirosis and other community-acquired forms of AKI.

We have previously challenged the generally held consensus that female sex is an independent risk factor for cardiac surgery-associated AKI and for aminoglycoside nephrotoxicity [18, 20]. In the present study, we sought to explore the relationship between sex and hospital-associated AKI (HAAKI) in greater detail by performing a systematic review and meta-analysis of studies published between January, 1978 and April, 2018 which report the sex-stratified incidence of HAAKI.

## Methods

### Search strategy and selection criteria

We conducted a systematic review and meta-analysis of the English literature to evaluate the reported incidence of acute kidney injury in hospitalized women versus hospitalized men. Our analysis was conducted according to the Preferred Reporting Items for Systematic Reviews and Meta-analyses protocol [21].

**Fig. 1:**
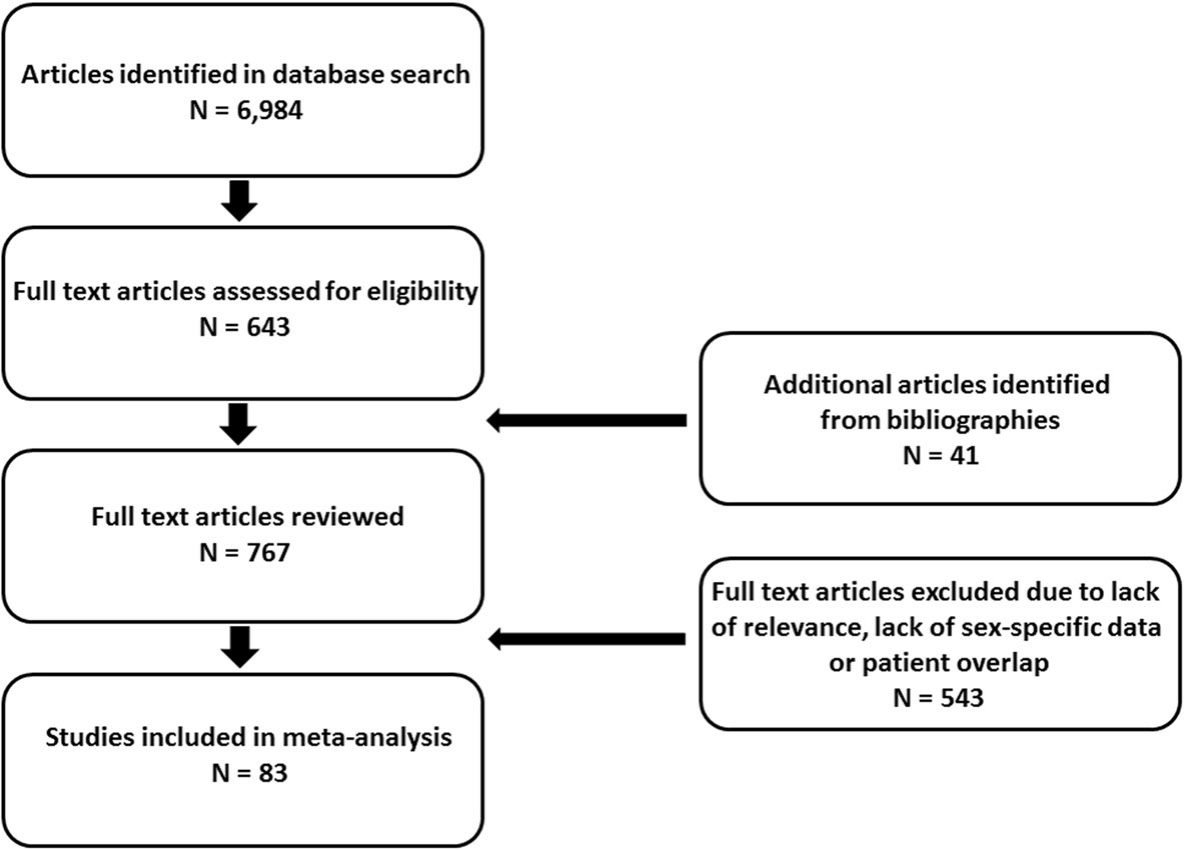
Flow diagram describing the identification of studies included in the meta-analysis

We searched PubMed for English-language articles published between January 1, 1978 and April 1, 2018. The following medical subject heading terms were used: male, female, sex, gender, acute kidney injury, and acute renal failure. EMBASE was also queried with the terms sex difference, acute kidney injury and acute renal failure. Titles and abstracts of articles found in the database search were reviewed to identify eligible studies. Full text versions of selected studies were analyzed in detail. We also examined the bibliographies of recovered articles for additional resources. Any case control or cohort study of 10,000 or more hospitalized patients in which the sex-stratified incidence of AKI was reported was eligible for inclusion (Fig. 1). To determine study quality, the studies were assessed using the Newcastle Ottawa Score for cohort and case control studies [22].

### Definition of AKI

Hospital-associated AKI was defined as AKI that developed in hospitalized patients. This definition included patients who developed AKI within the first 48 h of admission to the hospital (community-acquired AKI) and patients who developed AKI later during their hospital course (hospital-acquired). We accepted studies that defined AKI by investigator-created, creatinine-based criteria, Acute Kidney Injury Network (AKIN) criteria, Kidney Disease: Improving Global Outcomes (KDIGO) criteria, Risk, Injury, Failure, Loss of kidney function, End-stage kidney disease (RIFLE) criteria, or by the requirement for renal replacement therapy (AKI-D) [19, 23, 24].

### Data extraction

All studies were examined for duplication of data. Attention was given to the reporting clinical centers, years covered, and overlap with larger regional or national databases. In the case of overlap, a weighting factor was assigned to the smaller study that was inversely proportional to the degree of overlap. If the weighted number of patients fell below 10,000, the study was excluded. We also excluded studies with less than 25 AKI events among either of the sexes.

We separated the selected studies in to 2 groups. The first group included studies in which the investigators utilized multivariate analysis and reported adjusted odds ratios. The second group included studies in which unadjusted data was reported.

We analyzed separately studies restricted to patients who underwent radio-contrast procedures (percutaneous coronary interventions or computerized axial tomography) but which failed to specify whether the procedure was performed in an ambulatory care setting or was associated with an in-patient hospital stay. In this regard, most computerized axial tomography procedures are performed in an out-patient rather than in an in-patient setting and percutaneous coronary interventions have moved from an exclusively in-patient procedure to a predominantly ambulatory procedure over the last decade (0% ambulatory in 2009 to 77% ambulatory in 2015) [25].

### Statistical analysis

Data were analyzed using a random effects model with RevMan Version 5.3, The Cochrane Collaboration 2014. Meta-regression analysis and sub-group meta-analysis were performed with OpenMetaAnalyst 2016 [26].

## Results

### Adjusted analyses

**Fig. 2:**
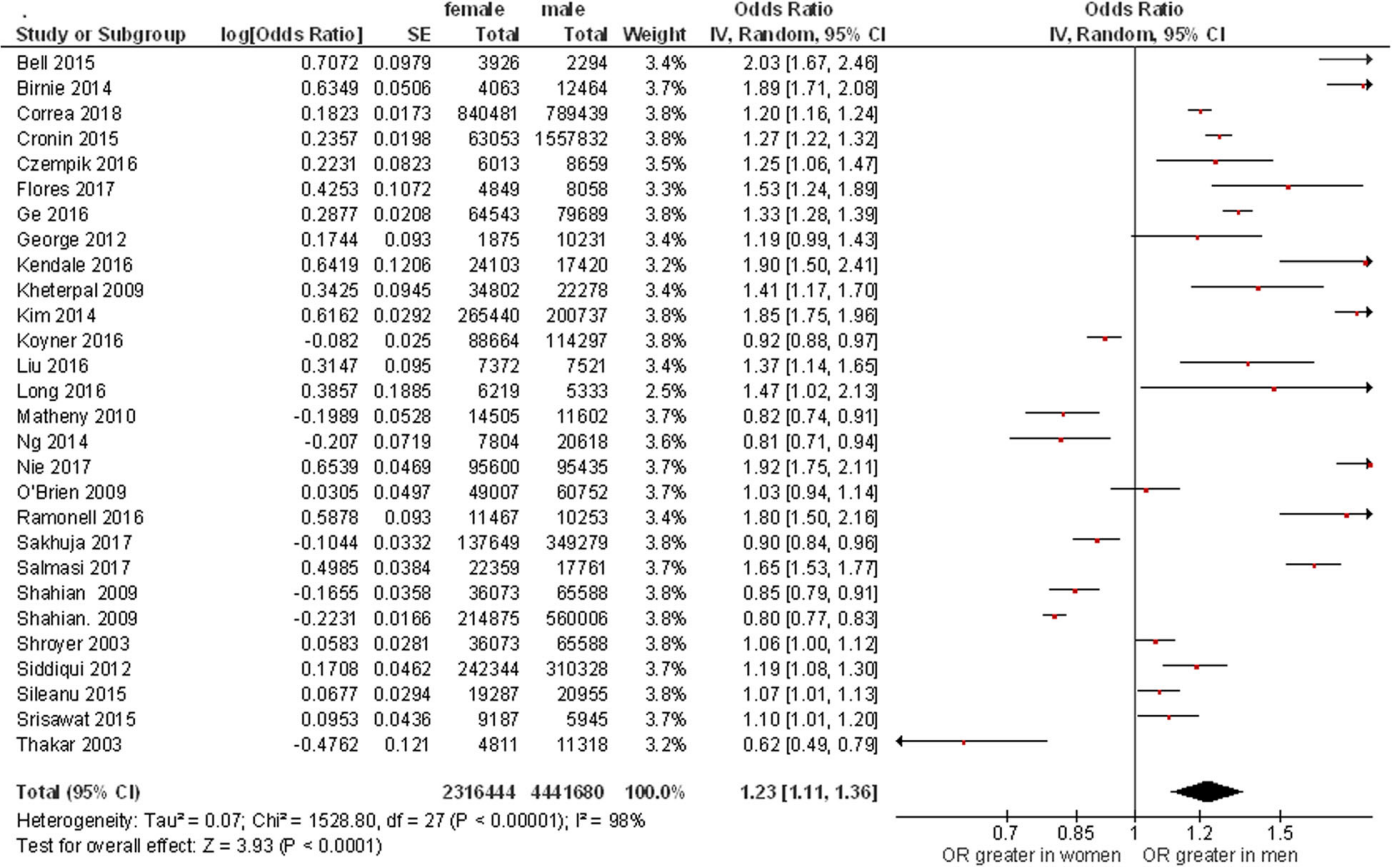
Meta-analysis of 28 studies that provided adjusted sex-stratified data regarding the incidence of hospital-associated AKI

Twenty-eight studies (6,758,124 patients; 2,313,202 women and 4,444,922 men) utilized multivariate analysis to assess risk factors for hospital-associated AKI and provided sex-stratified ORs (Fig. 2) [27–53]. Eight studies included only hospitalized patients who underwent cardiac surgery, 10 studies included only hospitalized patients who underwent predominantly non-cardiac surgery, 3 studies included only critically ill patients who received care in an intensive care unit, 6 studies included unselected hospitalized patients, whereas the remaining study included only hospitalized patients with a diagnosis of acute decompensated heart failure. AKI was defined by KDIGO criteria in 10 studies, by RIFLE criteria in 1 study, by AKIN criteria in 2 studies, by the need for renal replacement therapy in 7 studies, and by investigator-created, creatinine-based criteria in the remaining 8 studies. Nearly all studies that utilized RIFLE, AKIN or KDIGO criteria to define AKI relied solely on serum creatinine criteria rather than urine output criteria.

Meta-analysis of this cohort showed that men were significantly more likely to develop HAAKI than women (OR 1.23 (1.11,1.36), *n* = 28 studies, 6,758,124 patients).

We observed a high degree of statistical heterogeneity in the meta-analysis (I^2^ = 98.0%, *p* < 0.001). This is not surprising since AKI is not a single disease but instead represents a heterogeneous group of disorders characterized by an acute reduction in renal function. To evaluate the source of statistical heterogeneity, we performed a regression meta-analysis and subgroup analyses.

We found that statistical heterogeneity was related to the criteria used to select the study cohort and to the criteria used to define AKI, but was not related to year of publication, number of AKI events or total number of patients studied. The association of male sex with the development of AKI was strongest among studies restricted to patients who underwent predominantly non-cardiac surgery (OR 1.56 (1.37,1.77), *n* = 10 studies, 1,225418 patients, 606,881 women and 618,537 men). Male sex was also associated with AKI in studies of unselected hospitalized patients (OR 1.22 (1.01,1.49), *n* = 6 studies, 2,196,772 patients, 332,584 women and 2,196,772 men), and in studies of critically ill patients who received care in an intensive care unit (OR 1.10 (1.03,1.18), *n* = 3 studies, 70,046 patients, 34,487 women and 35,559 men). In contrast, cardiac surgery-associated AKI showed no sexual dimorphism (OR 0.95 (0.80,1.13), *n* = 8 studies, 1,635,968 patients, 490,355 women and 1,145,613 men).

The sex-stratified incidence of HAAKI also varied according to the criteria used to define AKI. Men were more likely to develop HAAKI than were women when AKI was identified by KDIGO criteria (OR 1.38 (1.19,1.59), *n* = 10 studies, 2,263,679 patients, 361,914 women and 1,901,765 men), and by AKIN criteria (OR 1.69 (1.52,1.88), *n* = 2 studies, 81,643 patients, 46,462 women and 35,181 men). There was no difference in the incidence of HAAKI between the sexes when AKI was identified by the need for renal replacement therapy (OR 1.05 (0.92, 1.10), *n* = 7 studies, 2,822,186 patients, 1,282,180 females and 1,540,006 men) or by investigator-created, creatinine-based criteria (1.19 (0.91, 1.55), *n* = 8 studies, 1,564,509 patients, 611,383 women and 953,126 men).

In a separate analysis, the incidence of AKI among adjusted studies of patients who underwent percutaneous coronary interventions or computerized axial tomography was equivalent in men and women (OR 1.05 (0.79,1.40), *n* = 3 studies, 1,087,879 patients, 347,811 women and 740,068 men) [54–56].

### Unadjusted analyses

**Fig. 3:**
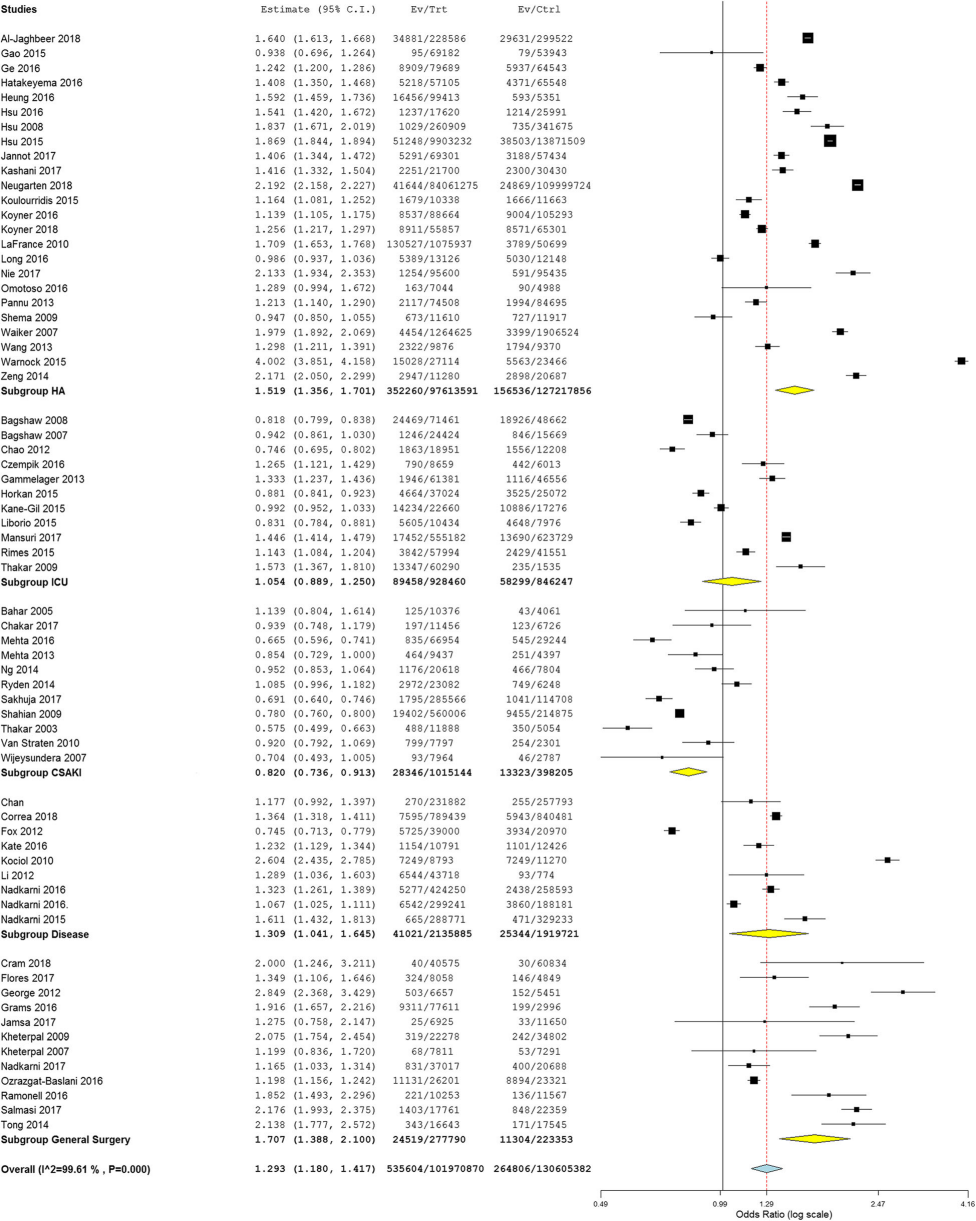
Subgroup meta-analysis of 68 studies that provided unadjusted sex-stratified data regarding the incidence of hospital-associated AKI. Abbreviations used: *ICU* Intensive care unit; *HA* Hospital-associated AK; *CSAKI* Cardiac surgery-associated AKI

**Fig. 4:**
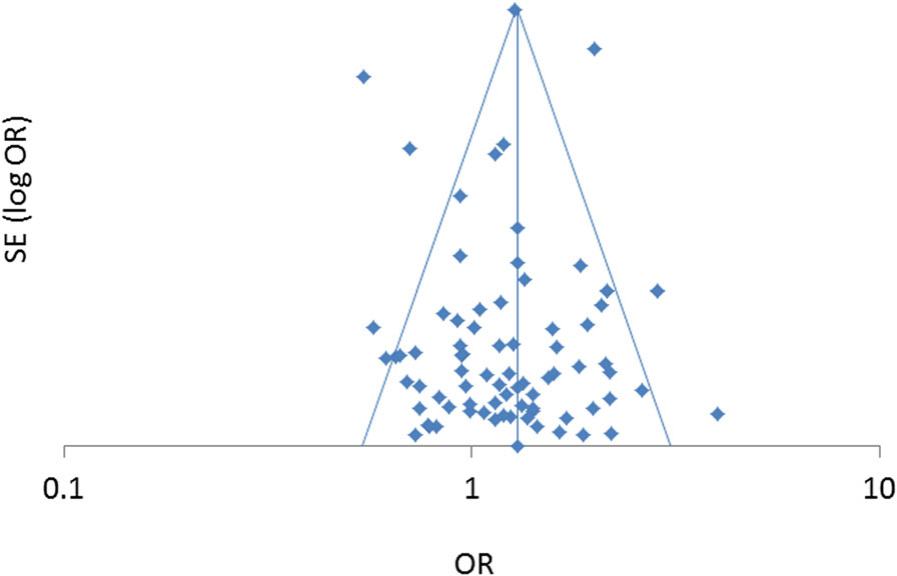
Funnel plot of studies that analyzed risk factors for hospital-associated AKI and provided sex-stratified odds ratios

The unadjusted cohort consisted of 68 studies which included 232,586,252 patients (130,605,382 women and 101,970,870 men (Figs. 3 and 4) [29, 31–34, 36, 38, 42, 43, 45, 47, 57–112]. Studies could be divided into 7 distinct categories. Twenty-four studies included unselected hospitalized patients, 11 studies included only hospitalized patients who underwent cardiac surgery, 13 studies included only hospitalized patients who underwent predominantly non-cardiac surgery, 11 studies included only critically ill patients who received care in an intensive care unit, whereas the remaining 9 studies included hospitalized patients selected based on their underlying disease (liver disease, cerebrovascular disease, human immunodeficiency virus infection, congestive heart failure, or atrial fibrillation). AKI was defined by RIFLE criteria in 5 studies, by AKIN criteria in 11 studies, by KDIGO criteria in 17 studies, by the need for renal replacement therapy in 20 studies, and by investigator-created, creatinine-based criteria in the remaining 15 studies. Nearly all studies that utilized RIFLE, AKIN or KDIGO criteria to define AKI relied solely on serum creatinine criteria rather than urine output criteria.

Meta-analysis of the entire cohort of unadjusted studies showed that men were significantly more likely to develop HAAKI than women (OR 1.29 (1.18,1.42), *n* = 68 studies, 232,586,252 patients). We observed a high degree of statistical heterogeneity in this analysis (I^2^ = 99.6%, *p* < 0.001). This is not surprising since AKI is not a single disease but instead represents a heterogeneous group of disorders characterized by an acute reduction in renal function. To evaluate the source of statistical heterogeneity, we performed a regression meta-analysis and subgroup analyses. We found that statistical heterogeneity was related to the criteria used to select the study cohort and to the criteria used to define AKI, but was not related to year of publication, number of AKI events or total number of patients.

The association of male sex with the development of AKI was strongest among studies reporting unadjusted data from patients undergoing predominantly non-cardiac surgery (OR 1.63 (1.34,1.97), *n* = 13 studies, 556,647 patients, 246,136 women and 310,511 men) and among studies of unselected hospitalized patients (OR 1.52 (1.34,1.70), *n* = 24 studies, 224,740,578 patients, 127,168,880 women and 97,571,698 men). Male sex was also associated with AKI among studies in which patients were selected based on a disease-specific diagnosis (1.31 (1.04,1.65), *n* = 9 studies, 4,055,606 patients, 1,919,721 women and 2,135,885 men). In contrast, among unadjusted studies of cardiac surgery-associated AKI, AKI was less frequent in men than in women (OR 0.82 (0.74, 0.91), *n* = 11 studies, 1,413,349 patients, 398,205 women and 1,015,144 men). The incidence of AKI among critically ill patients who received care in an intensive care unit was similar in men and women (OR 1.05 (0.89,1.25), *n* = 11 studies, 1,774,707 patients, 846,347 women and 928,460 men).

The unadjusted sex-stratified incidence of HAAKI also varied according to the criteria used to define AKI. Men were more likely to develop HAAKI than were women when AKI was identified by KDIGO criteria (OR 1.34 (1.20,1.51), *n* = 17 studies, 1,804,815 patients, 868,140 women and 936,675 men), or by the need for renal replacement therapy (OR 1.33 (1.17,1.50), *n* = 20 studies, 217,375,505 patients, 128,841,628 women and 98,533,877 men). In contrast, men were less likely to develop HAAKI than were women when AKI was identified by RIFLE criteria (OR 0.89 (0.82,0.96), *n* = 5 studies, 260,132 patients, 112,564 women and 157,568 men). There was no significant difference in the incidence of HAAKI between the sexes when AKI was identified by AKIN criteria (OR 1.23 (0.98,1.54), n = 11 studies, 1,783,778 patients, 286,062 women and 1,497,716 men) or by investigator-created, creatinine-based criteria (OR 1.37 (0.92, 2.03), *n* = 15 studies, 1,306,657 patients, 470,795 women and 835,862 men).

In a separate analysis of unadjusted studies, radio-contrast-induced AKI in patients undergoing computerized axial tomography or percutaneous coronary interventions was less frequent in men than in women (OR 0.79 (0.69,0.90), *n* = 9 studies, 1,516,807 patients, 478,719 women and 1,038,088 men).

## Discussion

Sexual dimorphism is a well-recognized feature of chronic progressive kidney disease [1]. Although less well recognized, sexual dimorphism has also been clearly established in AKI [2]. In contrast to CKD, where female sex is reno-protective, the direction of sexual dimorphism has been reported to be reversed in hospital-acquired AKI with female sex being associated with the development of AKI [19]. Moreover, female sex has been included as a risk factor in models developed to predict the risk of AKI associated with cardiac surgery, aminoglycoside nephrotoxicity, rhabdomyolysis and radio-contrast administration [15–18]. On the basis of these observations, the commentary to the KDIGO Clinical Practice Guideline for Acute Kidney Injury concludes that female sex is a risk factor for hospital-acquired AKI, while recognizing that male sex predominates in certain forms of community-acquired AKI. In the present study, we clearly show that it is male sex, not female sex, that is a risk factor for HAAKI, although we cannot determine whether this sexual dimorphism is driven by community-acquired or hospital-acquired AKI or both.

There is strong experimental basis to support our hypothesis that female sex is reno-protective in AKI [2–14, 20, 113]. Sexual dimorphism in AKI may be mediated by effects of sex hormones on cellular processes instrumental in the pathogenesis of AKI, analogous to our suggestion that sex hormones mediate sexual dimorphism in chronic kidney disease [1]. In experimental models of ischemic AKI, females show less severe renal functional impairment and less histologic damage after ischemia-reperfusion injury [2–14]. Numerous hypotheses have been proposed to explain these observations [2, 8, 113]. Sex-related differences in the generation of nitric oxide, in the synthesis and vascular response to endothelin-1, and in the renal hemodynamic response to angiotensin II have been demonstrated in experimental models and in human patients [2, 8]. Cellular responses to ischemia-reperfusion injury have also been shown to differ between the sexes. In response to ischemia-reperfusion, Na + -K+ ATPase enzyme activity is greater in females than in males and transcellular translocation of Na + -K+ ATPase is reduced [4]. Females subjected to ischemia-reperfusion injury maintain a reno-protective profile compared to their male counterparts with respect to heat shock protein HSP72, anti-oxidants such as superoxide dismutase, caspases and proteases involved in apoptosis, metalloproteinases such as meprin, inflammatory cytokines and members of signaling pathways that mediate pro-inflammatory responses [2–14, 113].

While our meta-analysis of adjusted studies demonstrated that, overall, female sex was associated with protection from HAAKI, our subgroup analysis revealed a relationship between the etiology of HAAKI and the presence or absence of sexual dimorphism. This is not surprising insofar as AKI is not a single disease but instead represents a heterogeneous group of disorders characterized by an acute reduction in renal function.

The association between female sex and protection from HAAKI was stronger among studies of hospitalized patients who underwent non-cardiac surgery than in the entire cohort of adjusted studies. Studies of critically ill patients receiving care in an intensive care unit and studies of unselected hospitalized patients also showed a higher incidence of AKI in men than in women. In this regard, unselected hospitalized patients better reflect the true relationship between sex and HAAKI as compared to studies in which patients were selected based on the etiology of AKI. In contrast, among studies of cardiac surgery-associated AKI, our meta-analysis demonstrated no difference between the sexes.

We have previously suggested that the association between female sex and cardiac surgery-associated AKI in unadjusted analyses reflects the greater burden of preexisting comorbidities among women undergoing cardiac surgery and does not indicate a greater intrinsic susceptibility of women to develop AKI under these circumstances [18]. This conclusion is reinforced by our demonstration in the present study that the sexual dimorphism associated with cardiac surgery-associated AKI in unadjusted analyses disappeared after adjustment for confounding factors.

It has been repeatedly demonstrated in unadjusted analyses and accepted by most authorities, including the commentary to the KDIGO Clinical Practice Guideline for AKI, that the incidence of contrast-induced nephropathy is greater in women than in men. However, some investigators have suggested that the association of contrast-induced nephropathy with female sex may merely reflects a higher dose of contrast administered to women compared to men [18]. Women generally have a lower body surface area than men, and accordingly the volume of administered contrast, when expressed as the volume of contrast administered per body surface area, has frequently been reported to be greater in women than in men. This hypothesis is consistent with our data which show that female sex was associated with contrast-induced nephropathy in unadjusted analyses, but that this association did not survive multivariate analysis.

We were surprised to find that only a modest, albeit significant, association of male sex with HAAKI in adjusted analyses of critically ill patients requiring care in an intensive care unit. Ischemic acute tubular necrosis is frequently the etiology of AKI in this setting and it is this form of renal injury that is most analogous to experimental ischemia-reperfusion injury, a model in which the reno-protection afforded by female sex is most robust [2–14].

A major limitation of our analysis relates to the inherent difficulty in defining AKI in men relative to women in light of sex-related differences in creatinine kinetics and the relationship of these differences to established criteria that define AKI. Waiker and Bonventre [114] assessed creatinine kinetics in patients with underlying chronic kidney disease and superimposed AKI. They identified differences in the sensitivity of absolute increases in serum creatinine levels versus relative increases in serum creatinine levels in identifying AKI in this population. They also emphasized the importance of the observation time in detecting threshold changes in serum creatinine levels. These observations are also relevant to comparisons of AKI incidence in men versus women. Since differences in the rate of generation and elimination of creatinine and in its volume of distribution exist between men and women with AKI, different criteria to define AKI might result in different sex-stratified incidence rates. Where AKI is defined by a percent change in the level of serum creatinine, the absolute change in creatinine needed to qualify as an AKI event is lower in women than in men since women generally have lower baseline serum creatinine levels. In contrast, where AKI is defined by an absolute increase in serum creatinine level, the percent change is serum creatinine required to qualify as an AKI event is greater in women than in men.

Also relevant to this issue are data reported by Srisawat et al. [52], which showed that the incidence of AKI was greater in men than in women when KDIGO criteria were used to define AKI, but that sex-related differences in the incidence of AKI disappeared when RIFLE criteria were used. These findings suggest that KDIGO criteria identify relatively more men than women with AKI compared to RIFLE criteria. Thus, it is possible that use of RIFLE criteria to define AKI, relative to KDIGO criteria, may mask the effect on female sex on the incidence of AKI, or conversely, that use of KDIGO criteria may magnify the effect. Consistent with this suggestion, our subgroup analysis shows that female sex was more likely to be associated with protection from AKI in those studies which utilized KDIGO criteria than in those that utilized RIFLE criteria. However, this conclusion is limited by the fact that our analysis, unlike the Srisawat data [52], compares outcomes based on differing definitions of AKI among different studies but not within an individual study.

We did not include in our meta-analysis 24 studies which utilized diagnosis codes to identify patients with non-dialysis-requiring AKI in the absence of corroborating biochemical data. Although Grams et al. [115] found a similar sensitivity and specificity for diagnosis codes in identifying AKI in men versus women, Waikar et al. [116] reported that the sensitivity was greater in men than in women. Were Waikar’s data to apply, any conclusions about the relationship between sex and AKI identified by diagnosis codes would be placed in serious jeopardy. Incidentally, the incidence of AKI was greater in men than in women in nearly all of these studies.

In contrast, we included studies that relied on AKI-D data identified by diagnosis and procedure codes. Numerous studies have established the high sensitivity, specificity, positive predictive value and negative predictive value of diagnostic codes to identify AKI-D in a variety of administrative databases [115–119]. These indices generally exceeded 90% in all studies except that reported by Grams et al. [115]. Not only do diagnostic codes to identify AKI-D have a greater accuracy than those to identify AKI, they are also unlikely to be subject to miscoding based on the sex of the patient. Yet the fact remains that, despite the objective basis for dialysis coding, the actual decision to initiate dialysis by the clinician is a subjective one.

We recently performed a systematic review of dialysis practices in AKI and found no evidence that dialysis is initiated more often or earlier in men than in women with AKI of identical severity [120]. In fact, data exist to indicate the opposite, i.e. that dialysis is more aggressively pursued in women than in men despite identical severity of AKI. After propensity score matching of patients with AKI, Wilson et al. [121] reported that dialysis was more likely to be initiated in women than in men. Similarly, Chou et al. [122] utilized propensity matching of patients with sepsis and AKI treated in surgical intensive care units and found that female sex was associated with earlier initiation of dialysis. Moreover, data from the North American Consortium for the Study of End-Stage Liver Disease indicates that hospitalized cirrhotic women are nearly twice as likely as men to receive renal replacement therapy despite similar median delta creatinine levels [123]. Thus, these studies suggest that the subjectivity inherent in the decision to initiate dialysis creates a bias that operates counter to our hypothesis, thereby strengthening our conclusion that the incidence of severe AKI requiring RRT is more common in men than in women.

## Conclusions

A meta-analysis of studies providing sex-stratified incidence of HAAKI demonstrates that female sex is associated with protection from AKI. This finding undermines the established belief that female sex is a significant risk factor for AKI. On the contrary, and consistent with observations in animal models, it is male sex that is associated with HAAKI.

## Abbreviations

AKI: Acute kidney injury
AKI-D: Acute kidney injury requiring dialysis
AKIN: Acute Kidney Injury Network
HAAKI: Hospital-associated acute kidney injury
KDIGO: Kidney Disease: Improving Global Outcomes
RIFLE: Risk, Injury, Failure, Loss of kidney function, End-stage kidney disease (RIFLE)
RRT: Renal replacement therapy

## References

[1] S.R. NJS, Golestaneh L, Amsterdam BBM (2008). Gender and kidney disease. *Brenner and Rector's The Kidney.* Edn.

[2] Hutchens MP, Dunlap J, Hurn PD, Jarnberg PO (2008). Renal ischemia: does sex matter?. Anesth Analg.

[3] Fekete A, Vannay A, Ver A, Rusai K, Muller V, Reusz G, Tulassay T, Szabo AJ (2006). Sex differences in heat shock protein 72 expression and localization in rats following renal ischemia-reperfusion injury. Am J Physiol Renal Physiol.

[4] Fekete A, Vannay A, Ver A, Vasarhelyi B, Muller V, Ouyang N, Reusz G, Tulassay T, Szabo AJ (2004). Sex differences in the alterations of Na(+), K(+)-ATPase following ischaemia-reperfusion injury in the rat kidney. J Physiol.

[5] Hutchens MP, Fujiyoshi T, Komers R, Herson PS, Anderson S (2012). Estrogen protects renal endothelial barrier function from ischemia-reperfusion in vitro and in vivo. Am J Physiol Renal Physiol.

[6] Kang KP, Lee JE, Lee AS, Jung YJ, Kim D, Lee S, Hwang HP, Kim W, Park SK (2014). Effect of gender differences on the regulation of renal ischemia-reperfusion-induced inflammation in mice. Mol Med Rep.

[7] Kher A, Meldrum KK, Wang M, Tsai BM, Pitcher JM, Meldrum DR (2005). Cellular and molecular mechanisms of sex differences in renal ischemia-reperfusion injury. Cardiovasc Res.

[8] Metcalfe PD, Meldrum KK (2006). Sex differences and the role of sex steroids in renal injury. J Urol.

[9] Muller V, Losonczy G, Heemann U, Vannay A, Fekete A, Reusz G, Tulassay T, Szabo AJ (2002). Sexual dimorphism in renal ischemia-reperfusion injury in rats: possible role of endothelin. Kidney Int.

[10] Park KM, Kim JI, Ahn Y, Bonventre AJ, Bonventre JV (2004). Testosterone is responsible for enhanced susceptibility of males to ischemic renal injury. J Biol Chem.

[11] Rodriguez F, Nieto-Ceron S, Fenoy FJ, Lopez B, Hernandez I, Martinez RR, Soriano MJ, Salom MG (2010). Sex differences in nitrosative stress during renal ischemia. Am J Physiol Regul Integr Comp Physiol.

[12] Satake A, Takaoka M, Nishikawa M, Yuba M, Shibata Y, Okumura K, Kitano K, Tsutsui H, Fujii K, Kobuchi S (2008). Protective effect of 17beta-estradiol on ischemic acute renal failure through the PI3K/Akt/eNOS pathway. Kidney Int.

[13] Takayama J, Takaoka M, Sugino Y, Yamamoto Y, Ohkita M, Matsumura Y (2007). Sex difference in ischemic acute renal failure in rats: approach by proteomic analysis. Biol Pharm Bull.

[14] Tanaka R, Tsutsui H, Kobuchi S, Sugiura T, Yamagata M, Ohkita M, Takaoka M, Yukimura T, Matsumura Y (2012). Protective effect of 17beta-estradiol on ischemic acute kidney injury through the renal sympathetic nervous system. Eur J Pharmacol.

[15] Mehran R, Aymong ED, Nikolsky E, Lasic Z, Iakovou I, Fahy M, Mintz GS, Lansky AJ, Moses JW, Stone GW (2004). A simple risk score for prediction of contrast-induced nephropathy after percutaneous coronary intervention: development and initial validation. J Am Coll Cardiol.

[16] Moore RD, Smith CR, Lipsky JJ, Mellits ED, Lietman PS (1984). Risk factors for nephrotoxicity in patients treated with aminoglycosides. Ann Intern Med.

[17] McMahon GM, Zeng X, Waikar SS (2013). A risk prediction score for kidney failure or mortality in rhabdomyolysis. JAMA Intern Med.

[18] Neugarten J, Sandilya S, Singh B, Golestaneh L (2016). Sex and the risk of AKI following cardio-thoracic surgery: a meta-analysis. Clin J Am Soc Nephrol.

[19] KDIGO Clinical Practice Guideline for Acute Kidney Injury. Kidney Int 2012, 2(Supplement 1):1–138; Online Appendices A-F.

[20] Neugarten J, Golestaneh L (2016). The effect of gender on aminoglycoside-associated nephrotoxicity. Clin Nephrol.

[21] Hutton B, Salanti G, Caldwell DM, Chaimani A, Schmid CH, Cameron C, Ioannidis JP, Straus S, Thorlund K, Jansen JP (2015). The PRISMA extension statement for reporting of systematic reviews incorporating network meta-analyses of health care interventions: checklist and explanations. Ann Intern Med.

[22] Wells GA SB, O’Connell D, Peterson J, Welch V, Losos M, Tugwell P:: The Newcastle-Ottawa Scale (NOS) for assessing the quality of nonrandomised studies in meta-analyses. *[*http://www.ohrica/programs/clinical_epidemiology/oxfordasp].

[23] Bellomo R, Ronco C, Kellum JA, Mehta RL, Palevsky P (2004). Acute Dialysis quality initiative w: acute renal failure - definition, outcome measures, animal models, fluid therapy and information technology needs: the second international consensus conference of the acute Dialysis quality initiative (ADQI) group. Crit Care.

[24] Mehta RL, Kellum JA, Shah SV, Molitoris BA, Ronco C, Warnock DG, Levin A (2007). Acute kidney injury N: acute kidney injury network: report of an initiative to improve outcomes in acute kidney injury. Crit Care.

[25] Valle JA, McCoy LA, Maddox TM, Rumsfeld JS, Ho PM, Casserly IP, Nallamothu BK, Roe MT, Tsai TT, Messenger JC. Longitudinal risk of adverse events in patients with acute kidney injury after percutaneous coronary intervention: insights from the National Cardiovascular Data Registry. Circ Cardiovasc Interv. 2017;10(4). 10.1161/CIRCINTERVENTIONS.116.004439 28404621

[26] Wallace BC, Schmid CH, Lau J, Trikalinos TA (2009). Meta-analyst: software for meta-analysis of binary, continuous and diagnostic data. BMC Med Res Methodol.

[27] Bell S, Dekker FW, Vadiveloo T, Marwick C, Deshmukh H, Donnan PT, Van Diepen M (2015). Risk of postoperative acute kidney injury in patients undergoing orthopaedic surgery--development and validation of a risk score and effect of acute kidney injury on survival: observational cohort study. BMJ.

[28] Birnie K, Verheyden V, Pagano D, Bhabra M, Tilling K, Sterne JA, Murphy GJ, Collaborators UAiCS (2014). Predictive models for kidney disease: improving global outcomes (KDIGO) defined acute kidney injury in UK cardiac surgery. Crit Care.

[29] Correa Ashish, Patel Achint, Chauhan Kinsuk, Shah Harshil, Saha Aparna, Dave Mihir, Poojary Priti, Mishra Abhishek, Annapureddy Narender, Dalal Shaman, Konstantinidis Ioannis, Nimma Renu, Agarwal Shiv Kumar, Chan Lili, Nadkarni Girish, Pinney Sean (2018). National Trends and Outcomes in Dialysis-Requiring Acute Kidney Injury in Heart Failure: 2002–2013. Journal of Cardiac Failure.

[30] Cronin RM, VanHouten JP, Siew ED, Eden SK, Fihn SD, Nielson CD, Peterson JF, Baker CR, Ikizler TA, Speroff T (2015). National Veterans Health Administration inpatient risk stratification models for hospital-acquired acute kidney injury. J Am Med Inform Assoc.

[31] Czempik P, Ciesla D, Knapik P, Krzych LJ (2016). Risk factors of acute kidney injury requiring renal replacement therapy based on regional registry data. Anaesthesiol Intensive Ther.

[32] Flores E, Lewinger JP, Rowe VL, Woo K, Weaver FA, Shavelle D, Clavijo L, Garg PK (2017). Increased risk of mortality after lower extremity bypass in individuals with acute kidney injury in the vascular quality initiative. J Vasc Surg.

[33] Ge S, Nie S, Liu Z, Chen C, Zha Y, Qian J, Liu B, Teng S, Xu A, Bin W (2016). Epidemiology and outcomes of acute kidney injury in elderly chinese patients: a subgroup analysis from the EACH study. BMC Nephrol.

[34] George TJ, Arnaoutakis GJ, Beaty CA, Pipeling MR, Merlo CA, Conte JV, Shah AS (2012). Acute kidney injury increases mortality after lung transplantation. Ann Thorac Surg.

[35] Kendale SM, Lapis PN, Melhem SM, Blitz JD (2016). The association between pre-operative variables, including blood pressure, and postoperative kidney function. Anaesthesia.

[36] Kheterpal S, Tremper KK, Heung M, Rosenberg AL, Englesbe M, Shanks AM, Campbell DA (2009). Development and validation of an acute kidney injury risk index for patients undergoing general surgery: results from a national data set. Anesthesiology.

[37] Kim M, Brady JE, Li G (2014). Variations in the risk of acute kidney injury across intraabdominal surgery procedures. Anesth Analg.

[38] Koyner JL, Adhikari R, Edelson DP, Churpek MM (2016). Development of a multicenter Ward-based AKI prediction model. Clin J Am Soc Nephrol.

[39] Liu X, Ye Y, Mi Q, Huang W, He T, Huang P, Xu N, Wu Q, Wang A, Li Y (2016). A predictive model for assessing surgery-related acute kidney injury risk in hypertensive patients: a retrospective cohort study. PLoS One.

[40] Long TE, Helgason D, Helgadottir S, Palsson R, Gudbjartsson T, Sigurdsson GH, Indridason OS, Sigurdsson MI (2016). Acute kidney injury after abdominal surgery: incidence, risk factors, and outcome. Anesth Analg.

[41] Matheny ME, Miller RA, Ikizler TA, Waitman LR, Denny JC, Schildcrout JS, Dittus RS, Peterson JF (2010). Development of inpatient risk stratification models of acute kidney injury for use in electronic health records. Med Decis Mak.

[42] Ng SY, Sanagou M, Wolfe R, Cochrane A, Smith JA, Reid CM (2014). Prediction of acute kidney injury within 30 days of cardiac surgery. J Thorac Cardiovasc Surg.

[43] Nie S, Feng Z, Tang L, Wang X, He Y, Fang J, Li S, Yang Y, Mao H, Jiao J (2017). Risk factor analysis for AKI including laboratory indicators: a Nationwide multicenter study of hospitalized patients. Kidney Blood Press Res.

[44] O'Brien SM, Shahian DM, Filardo G, Ferraris VA, Haan CK, Rich JB, Normand SL, DeLong ER, Shewan CM, Dokholyan RS (2009). The Society of Thoracic Surgeons 2008 cardiac surgery risk models: part 2--isolated valve surgery. Ann Thorac Surg.

[45] Ramonell KM, Fang S, Perez SD, Srinivasan JK, Sullivan PS, Galloway JR, Staley CA, Lin E, Sharma J, Sweeney JF (2016). Development and validation of a risk calculator for renal complications after colorectal surgery using the National Surgical Quality Improvement Program Participant use Files. Am Surg.

[46] Sakhuja A, Kashani K, Schold J, Cheungpasitporn W, Soltesz E, Demirjian S (2017). Hospital procedure volume does not predict acute kidney injury after coronary artery bypass grafting-a nationwide study. Clin Kidney J.

[47] Salmasi V, Maheshwari K, Yang D, Mascha EJ, Singh A, Sessler DI, Kurz A (2017). Relationship between intraoperative hypotension, defined by either reduction from baseline or absolute thresholds, and acute kidney and myocardial injury after noncardiac surgery: a retrospective cohort analysis. Anesthesiology.

[48] Shahian DM, O'Brien SM, Filardo G, Ferraris VA, Haan CK, Rich JB, Normand SL, DeLong ER, Shewan CM, Dokholyan RS (2009). The Society of Thoracic Surgeons 2008 cardiac surgery risk models: part 3--valve plus coronary artery bypass grafting surgery. Ann Thorac Surg.

[49] Shroyer AL, Coombs LP, Peterson ED, Eiken MC, DeLong ER, Chen A, Ferguson TB, Grover FL, Edwards FH (2003). Society of Thoracic S: the Society of Thoracic Surgeons: 30-day operative mortality and morbidity risk models. Ann Thorac Surg.

[50] Siddiqui NF, Coca SG, Devereaux PJ, Jain AK, Li L, Luo J, Parikh CR, Paterson M, Philbrook HT, Wald R (2012). Secular trends in acute dialysis after elective major surgery--1995 to 2009. CMAJ.

[51] Sileanu FE, Murugan R, Lucko N, Clermont G, Kane-Gill SL, Handler SM, Kellum JA (2015). AKI in low-risk versus high-risk patients in intensive care. Clin J Am Soc Nephrol.

[52] Srisawat N, Sileanu FE, Murugan R, Bellomod R, Calzavacca P, Cartin-Ceba R, Cruz D, Finn J, Hoste EE, Kashani K (2015). Variation in risk and mortality of acute kidney injury in critically ill patients: a multicenter study. Am J Nephrol.

[53] Thakar CV, Liangos O, Yared JP, Nelson D, Piedmonte MR, Hariachar S, Paganini EP (2003). ARF after open-heart surgery: influence of gender and race. Am J Kidney Dis.

[54] Aubry P, Brillet G, Catella L, Schmidt A, Benard S (2016). Outcomes, risk factors and health burden of contrast-induced acute kidney injury: an observational study of one million hospitalizations with image-guided cardiovascular procedures. BMC Nephrol.

[55] Brown JR, DeVries JT, Piper WD, Robb JF, Hearne MJ, Ver Lee PM, Kellet MA, Watkins MW, Ryan TJ, Silver MT (2008). Serious renal dysfunction after percutaneous coronary interventions can be predicted. Am Heart J.

[56] Khanal S, Attallah N, Smith DE, Kline-Rogers E, Share D, O'Donnell MJ, Moscucci M (2005). Statin therapy reduces contrast-induced nephropathy: an analysis of contemporary percutaneous interventions. Am J Med.

[57] Al-Jaghbeer M, Dealmeida D, Bilderback A, Ambrosino R, Kellum JA (2018). Clinical decision support for in-hospital AKI. J Am Soc Nephrol.

[58] Bagshaw SM, George C, Bellomo R, Committee ADM (2007). Changes in the incidence and outcome for early acute kidney injury in a cohort of Australian intensive care units. Crit Care.

[59] Bagshaw SM, George C, Bellomo R, Committee ADM (2008). Early acute kidney injury and sepsis: a multicentre evaluation. Crit Care.

[60] Bahar I, Akgul A, Ozatik MA, Vural KM, Demirbag AE, Boran M, Tasdemir O (2005). Acute renal failure following open heart surgery: risk factors and prognosis. Perfusion.

[61] Briggs A, Havens JM, Salim A, Christopher KB. Acute kidney injury predicts mortality in emergency general surgery patients. Am J Surg. 2018. 10.1016/j.amjsurg.2018.03.015 29615192

[62] Chaker Z, Badhwar V, Alqahtani F, Aljohani S, Zack CJ, Holmes DR, Rihal CS, Alkhouli M. Sex differences in the utilization and outcomes of surgical aortic valve replacement for severe aortic stenosis. J Am Heart Assoc. 2017;6(9). 10.1161/JAHA.117.006370 PMC5634288 28935681

[63] Chan L, Mehta S, Chauhan K, Poojary P, Patel S, Pawar S, Patel A, Correa A, Patel S, Garimella PS, et al. National Trends and impact of acute kidney injury requiring hemodialysis in hospitalizations with atrial fibrillation. J Am Heart Assoc. 2016;5(12). 10.1161/JAHA.116.004509 PMC5210405 27998917

[64] Chao CT, Hou CC, Wu VC, Lu HM, Wang CY, Chen L, Kao TW (2012). The impact of dialysis-requiring acute kidney injury on long-term prognosis of patients requiring prolonged mechanical ventilation: nationwide population-based study. PLoS One.

[65] Cram P, Hawker G, Matelski J, Ravi B, Pugely A, Gandhi R, Jackson T (2018). Disparities in knee and hip arthroplasty outcomes: an observational analysis of the ACS-NSQIP clinical registry. J Racial Ethn Health Disparities.

[66] Fox CS, Muntner P, Chen AY, Alexander KP, Roe MT, Wiviott SD (2012). Short-term outcomes of acute myocardial infarction in patients with acute kidney injury: a report from the national cardiovascular data registry. Circulation.

[67] Gammelager H, Christiansen CF, Johansen MB, Tonnesen E, Jespersen B, Sorensen HT (2013). Five-year risk of end-stage renal disease among intensive care patients surviving dialysis-requiring acute kidney injury: a nationwide cohort study. Crit Care.

[68] Gao J, Chen M, Wang X, Wang H, Zhuo L (2015). Risk factors and prognosis of acute kidney injury in adult hospitalized patients: a two-year outcome. Minerva Urol Nefrol.

[69] Grams ME, Sang Y, Coresh J, Ballew S, Matsushita K, Molnar MZ, Szabo Z, Kalantar-Zadeh K, Kovesdy CP (2016). Acute kidney injury after major surgery: a retrospective analysis of veterans health administration data. Am J Kidney Dis.

[70] Hatakeyama Y, Horino T, Kataoka H, Matsumoto T, Ode K, Shimamura Y, Ogata K, Inoue K, Taniguchi Y, Terada Y (2017). Incidence of acute kidney injury among patients with chronic kidney disease: a single-center retrospective database analysis. Clin Exp Nephrol.

[71] Heung M, Steffick DE, Zivin K, Gillespie BW, Banerjee T, Hsu CY, Powe NR, Pavkov ME, Williams DE, Saran R (2016). Acute kidney injury recovery pattern and subsequent risk of CKD: an analysis of veterans health administration data. Am J Kidney Dis.

[72] Horkan CM, Purtle SW, Mendu ML, Moromizato T, Gibbons FK, Christopher KB (2015). The association of acute kidney injury in the critically ill and postdischarge outcomes: a cohort study. Crit Care Med.

[73] Hsu CY, Hsu RK, Yang J, Ordonez JD, Zheng S, Go AS (2016). Elevated BP after AKI. J Am Soc Nephrol.

[74] Hsu CY, Ordonez JD, Chertow GM, Fan D, McCulloch CE, Go AS (2008). The risk of acute renal failure in patients with chronic kidney disease. Kidney Int.

[75] Hsu RK, McCulloch CE, Heung M, Saran R, Shahinian VB, Pavkov ME, Burrows NR, Powe NR, Hsu CY, Centers for disease C (2016). Exploring potential reasons for the temporal trend in Dialysis-requiring AKI in the United States. Clin J Am Soc Nephrol.

[76] Jamsa P, Jamsen E, Lyytikainen LP, Kalliovalkama J, Eskelinen A, Oksala N (2017). Risk factors associated with acute kidney injury in a cohort of 20,575 arthroplasty patients. Acta Orthop.

[77] Jannot AS, Burgun A, Thervet E, Pallet N (2017). The diagnosis-wide landscape of hospital-acquired AKI. Clin J Am Soc Nephrol.

[78] Kane-Gill SL, Sileanu FE, Murugan R, Trietley GS, Handler SM, Kellum JA (2015). Risk factors for acute kidney injury in older adults with critical illness: a retrospective cohort study. Am J Kidney Dis.

[79] Kashani K, Shao M, Li G, Williams AW, Rule AD, Kremers WK, Malinchoc M, Gajic O, Lieske JC (2017). No increase in the incidence of acute kidney injury in a population-based annual temporal trends epidemiology study. Kidney Int.

[80] Kate RJ, Perez RM, Mazumdar D, Pasupathy KS, Nilakantan V (2016). Prediction and detection models for acute kidney injury in hospitalized older adults. BMC Med Inform Decis Mak.

[81] Kheterpal S, Tremper KK, Englesbe MJ, O'Reilly M, Shanks AM, Fetterman DM, Rosenberg AL, Swartz RD (2007). Predictors of postoperative acute renal failure after noncardiac surgery in patients with previously normal renal function. Anesthesiology.

[82] Kociol RD, Greiner MA, Hammill BG, Phatak H, Fonarow GC, Curtis LH, Hernandez AF (2010). Long-term outcomes of medicare beneficiaries with worsening renal function during hospitalization for heart failure. Am J Cardiol.

[83] Koulouridis I, Price LL, Madias NE, Jaber BL (2015). Hospital-acquired acute kidney injury and hospital readmission: a cohort study. Am J Kidney Dis.

[84] Koyner JL, Carey KA, Edelson DP, Churpek MM. The development of a machine learning inpatient acute kidney injury prediction model. Crit Care Med. 2018. 10.1097/CCM.0000000000003123 29596073

[85] Lafrance JP, Miller DR (2010). Defining acute kidney injury in database studies: the effects of varying the baseline kidney function assessment period and considering CKD status. Am J Kidney Dis.

[86] Li Y, Shlipak MG, Grunfeld C, Choi AI (2012). Incidence and risk factors for acute kidney injury in HIV infection. Am J Nephrol.

[87] Liborio AB, Leite TT, Neves FM, Teles F, Bezerra CT (2015). AKI complications in critically ill patients: association with mortality rates and RRT. Clin J Am Soc Nephrol.

[88] Long TE, Sigurdsson MI, Sigurdsson GH, Indridason OS (2016). Improved long-term survival and renal recovery after acute kidney injury in hospitalized patients: a 20 year experience. Nephrology (Carlton).

[89] Mansuri U, Patel A, Shah H, Chauhan K, Poojary P, Saha A, Dave M, Hazra A, Mishra T, Annapureddy N (2017). Trends and outcomes of sepsis hospitalizations complicated by acute kidney injury requiring hemodialysis. J Crit Care.

[90] Mehta RH, Castelvecchio S, Ballotta A, Frigiola A, Bossone E, Ranucci M (2013). Association of gender and lowest hematocrit on cardiopulmonary bypass with acute kidney injury and operative mortality in patients undergoing cardiac surgery. Ann Thorac Surg.

[91] Mehta RH, Grab JD, O'Brien SM, Bridges CR, Gammie JS, Haan CK, Ferguson TB, Peterson ED (2006). Society of Thoracic Surgeons National Cardiac Surgery Database I: bedside tool for predicting the risk of postoperative dialysis in patients undergoing cardiac surgery. Circulation.

[92] Nadkarni GN, Chauhan K, Patel A, Saha A, Poojary P, Kamat S, Patel S, Ferrandino R, Konstantinidis I, Garimella PS (2017). Temporal trends of dialysis requiring acute kidney injury after orthotopic cardiac and liver transplant hospitalizations. BMC Nephrol.

[93] Nadkarni GN, Patel A, Simoes PK, Yacoub R, Annapureddy N, Kamat S, Konstantinidis I, Perumalswami P, Branch A, Coca SG (2016). Dialysis-requiring acute kidney injury among hospitalized adults with documented hepatitis C virus infection: a nationwide inpatient sample analysis. J Viral Hepat.

[94] Nadkarni GN, Patel AA, Konstantinidis I, Mahajan A, Agarwal SK, Kamat S, Annapureddy N, Benjo A, Thakar CV (2015). Dialysis requiring acute kidney injury in acute cerebrovascular accident hospitalizations. Stroke.

[95] Nadkarni GN, Simoes PK, Patel A, Patel S, Yacoub R, Konstantinidis I, Kamat S, Annapureddy N, Parikh CR, Coca SG (2016). National trends of acute kidney injury requiring dialysis in decompensated cirrhosis hospitalizations in the United States. Hepatol Int.

[96] Neugarten J, Golestaneh L, Kolhe NV (2018). Sex differences in acute kidney injury requiring dialysis. BMC Nephrol.

[97] Omotoso BA, Abdel-Rahman EM, Xin W, Ma JZ, Scully KW, Arogundade FA, Balogun RA (2016). Dialysis requirement, long-term major adverse cardiovascular events (MACE) and all-cause mortality in hospital acquired acute kidney injury (AKI): a propensity-matched cohort study. J Nephrol.

[98] Ozrazgat-Baslanti T, Thottakkara P, Huber M, Berg K, Gravenstein N, Tighe P, Lipori G, Segal MS, Hobson C, Bihorac A (2016). Acute and chronic kidney disease and cardiovascular mortality after major surgery. Ann Surg.

[99] Pannu N, James M, Hemmelgarn B, Klarenbach S, Alberta kidney disease N (2013). Association between AKI, recovery of renal function, and long-term outcomes after hospital discharge. Clin J Am Soc Nephrol.

[100] Rimes-Stigare C, Frumento P, Bottai M, Martensson J, Martling CR, Bell M (2015). Long-term mortality and risk factors for development of end-stage renal disease in critically ill patients with and without chronic kidney disease. Crit Care.

[101] Ryden L, Sartipy U, Evans M, Holzmann MJ (2014). Acute kidney injury after coronary artery bypass grafting and long-term risk of end-stage renal disease. Circulation.

[102] Sakhuja A, Kumar G, Gupta S, Mittal T, Taneja A, Nanchal RS (2015). Acute kidney injury requiring Dialysis in severe Sepsis. Am J Respir Crit Care Med.

[103] Shahian DM, O'Brien SM, Filardo G, Ferraris VA, Haan CK, Rich JB, Normand SL, DeLong ER, Shewan CM, Dokholyan RS (2009). The Society of Thoracic Surgeons 2008 cardiac surgery risk models: part 1--coronary artery bypass grafting surgery. Ann Thorac Surg.

[104] Shema L, Ore L, Geron R, Kristal B (2009). Hospital-acquired acute kidney injury in Israel. Isr Med Assoc J.

[105] Thakar CV, Christianson A, Freyberg R, Almenoff P, Render ML (2009). Incidence and outcomes of acute kidney injury in intensive care units: a veterans administration study. Crit Care Med.

[106] Tong BC, Kosinski AS, Burfeind WR, Onaitis MW, Berry MF, Harpole DH, D'Amico TA (2014). Sex differences in early outcomes after lung cancer resection: analysis of the Society of Thoracic Surgeons general thoracic database. J Thorac Cardiovasc Surg.

[107] van Straten AH, Hamad MA, van Zundert AA, Martens EJ, Schonberger JP, de Wolf AM (2010). Risk factors for deterioration of renal function after coronary artery bypass grafting. Eur J Cardiothorac Surg.

[108] Waikar SS, Curhan GC, Ayanian JZ, Chertow GM (2007). Race and mortality after acute renal failure. J Am Soc Nephrol.

[109] Wang HE, Jain G, Glassock RJ, Warnock DG (2013). Comparison of absolute serum creatinine changes versus kidney disease: improving global outcomes consensus definitions for characterizing stages of acute kidney injury. Nephrol Dial Transplant.

[110] Warnock DG, Powell TC, Donnelly JP, Wang HE (2015). Categories of hospital-associated acute kidney injury: time course of changes in serum creatinine values. Nephron.

[111] Wijeysundera DN, Karkouti K, Dupuis JY, Rao V, Chan CT, Granton JT, Beattie WS (2007). Derivation and validation of a simplified predictive index for renal replacement therapy after cardiac surgery. JAMA.

[112] Zeng X, McMahon GM, Brunelli SM, Bates DW, Waikar SS (2014). Incidence, outcomes, and comparisons across definitions of AKI in hospitalized individuals. Clin J Am Soc Nephrol.

[113] Dubey RK, Jackson EK (2001). Estrogen-induced cardiorenal protection: potential cellular, biochemical, and molecular mechanisms. Am J Physiol Renal Physiol.

[114] Waikar SS, Bonventre JV (2009). Creatinine kinetics and the definition of acute kidney injury. J Am Soc Nephrol.

[115] Grams ME, Waikar SS, MacMahon B, Whelton S, Ballew SH, Coresh J (2014). Performance and limitations of administrative data in the identification of AKI. Clin J Am Soc Nephrol.

[116] Waikar SS, Wald R, Chertow GM, Curhan GC, Winkelmayer WC, Liangos O, Sosa MA, Jaber BL (2006). Validity of international classification of diseases, ninth revision, clinical modification codes for acute renal failure. J Am Soc Nephrol.

[117] Quinn RR, Laupacis A, Austin PC, Hux JE, Garg AX, Hemmelgarn BR, Oliver MJ (2010). Using administrative datasets to study outcomes in dialysis patients: a validation study. Med Care.

[118] Romano PS, Mark DH (1994). Bias in the coding of hospital discharge data and its implications for quality assessment. Med Care.

[119] Vlasschaert ME, Bejaimal SA, Hackam DG, Quinn R, Cuerden MS, Oliver MJ, Iansavichus A, Sultan N, Mills A, Garg AX (2011). Validity of administrative database coding for kidney disease: a systematic review. Am J Kidney Dis.

[120] Blush J, Lei J, Ju W, Silbiger S, Pullman J, Neugarten J (2004). Estradiol reverses renal injury in Alb/TGF-beta1 transgenic mice. Kidney Int.

[121] Wilson FP, Yang W, Machado CA, Mariani LH, Borovskiy Y, Berns JS, Feldman HI (2014). Dialysis versus nondialysis in patients with AKI: a propensity-matched cohort study. Clin J Am Soc Nephrol.

[122] Chou YH, Huang TM, Wu VC, Wang CY, Shiao CC, Lai CF, Tsai HB, Chao CT, Young GH, Wang WJ (2011). Impact of timing of renal replacement therapy initiation on outcome of septic acute kidney injury. Crit Care.

[123] O'Leary JG, Wong F, Reddy KR, Garcia-Tsao G, Kamath PS, Biggins SW, Fallon MB, Subramanian RM, Maliakkal B, Thacker L (2017). Gender-specific differences in baseline, peak, and Delta serum creatinine: the NACSELD experience. Dig Dis Sci.

